# Divergent Impacts of Straw and Biochar on Organic Carbon Fraction Allocation and CO_2_ Emissions in Coastal Saline Soil

**DOI:** 10.3390/toxics14100917

**Published:** 2026-10-16

**Authors:** Weijia Li, Haiyang Pang, Liang Yu, Fan Wei, Peng Gao, Luqin Sun, Guanhong Wang, Jie Ji, Xiao Li

**Affiliations:** College of Ecology, Resources and Environment, Dezhou University, Dezhou 253023, China; weijiali@nwafu.edu.cn (W.L.); panghaiyang@dzu.edu.cn (H.P.); yuliang@dzu.edu.cn (L.Y.); 15064002733@139.com (F.W.); gaopeng@dzu.edu.cn (P.G.); slq6172006@126.com (L.S.); wangguanhong2015@163.com (G.W.); seasonchina@sina.com (J.J.)

**Keywords:** saline-alkali soil amelioration, microbial community composition, particulate organic carbon, mineral-associated organic carbon, carbon mineralization

## Abstract

How homologous straw and biochar alter soil carbon fractions and associated microbial processes in coastal saline soils remains unclear. A 120-day microcosm experiment compared reed (*Phragmites australis*) straw and its biochar at equal mass rates of 1%, 5%, and 9% of dry soil mass. Both amendments increased the soil organic carbon (SOC) and reduced electrical conductivity. At 9%, straw increased the particulate organic carbon share of SOC to 78.2%, decreased the bacterial oligotroph/copiotroph ratio from 1.82 to 0.86, and raised potential hydrolytic enzyme activities to 4.3–9.1 times the control levels. Across straw treatments, cumulative CO_2_ emissions were 375.0–1645.9% higher than in the unamended control. Biochar maintained operationally defined mineral-associated organic carbon dominance (62.1–68.0% of SOC), with weaker hydrolytic responses and cumulative CO_2_ emissions 58.0–102.2% above the control levels. Exploratory structural equation models showed that lower bacterial oligotroph/copiotroph ratios were associated with higher hydrolytic enzyme activity under straw. Under biochar, higher fungal Basidiomycota/Ascomycota ratios were associated with higher polyphenol oxidase activity. Both enzyme indicators were positively associated with cumulative CO_2_ emissions in their respective models. These findings demonstrate contrasting carbon fraction distributions and respiratory responses to homologous amendments, with implications for carbon management in coastal saline soils.

## 1. Introduction

Soil salinization is a pervasive constraint on agricultural productivity and soil functioning, with salt-affected soils now covering approximately 10.7% of the global land area [1]. Coastal saline agroecosystems are particularly vulnerable because osmotic stress suppresses microbial activity, limits plant-derived carbon inputs, and destabilizes soil aggregates, collectively restricting both the magnitude and the persistence of soil organic carbon (SOC) accumulation [2,3]. Organic amendments, including crop residue return and pyrolytic biochar, are widely promoted as strategies to replenish SOC and rehabilitate degraded coastal soils [4,5]. Yet a higher SOC concentration after amendment does not necessarily indicate greater carbon persistence [6], because the fate of introduced carbon depends on whether it is physically protected within soil aggregates and organo-mineral complexes or remains as unprotected particulate material vulnerable to rapid microbial mineralization [7,8]. This distinction has direct implications for whether organic amendment constitutes genuine carbon sequestration or merely a transient pulse of carbon that is rapidly respired back to the atmosphere.

The dual-carbon pool paradigm, which differentiates particulate organic carbon (POC) from mineral-associated organic carbon (MAOC), provides a process-based framework for understanding carbon persistence [7,9]. POC consists largely of partially depolymerized plant debris that receives minimal direct mineral protection, functioning as an active, short-lived pool that accelerates microbial turnover and nutrient cycling [7,10]. Conversely, MAOC is primarily formed via the sorption and organo-mineral entrainment of microbially derived metabolites and necromass onto silt and clay-sized fractions, imparting decadal persistence and resistance to oxidation [9]. In coastal saline soils, this framework is subject to additional constraints imposed by salinity stress. Salinity-induced colloidal dispersion, displacement of Ca^2+^ and Mg^2+^ by Na^+^, and limited reactive clay surfaces can weaken organo-mineral association, leaving exogenous carbon more likely to remain in POC or to be directly mineralized [3,11]. Therefore, the application of straw and biochar to ameliorate saline soils may not only increase the soil organic carbon content, but also alter the partitioning of exogenous carbon between POC and MAOC [12,13]. Straw is rich in hydrolyzable carbohydrates and enters soil mainly as coarse particulate residues, which may preferentially enter POC and turn over rapidly under microbial attack [14,15]. In contrast, oxygen-limited pyrolysis reconfigures plant tissues into fused polycyclic aromatic structures, reducing immediate substrate accessibility; physical microfragmentation and electrostatic bridging or ligand exchange between surface oxygen-containing functional groups and Ca^2+^/Mg^2+^ may favor incorporation into MAOC or the formation of stable organo-mineral complexes [16,17,18]. Comparative studies have shown that straw and biochar differ in their effects on POC/MAOC dynamics and SOC sequestration [13]. However, these findings are largely derived from non-saline soils or non-homologous feedstocks. Whether homologous straw and biochar lead to divergent POC/MAOC partitioning in coastal saline soils remains untested.

**Figure 1:**
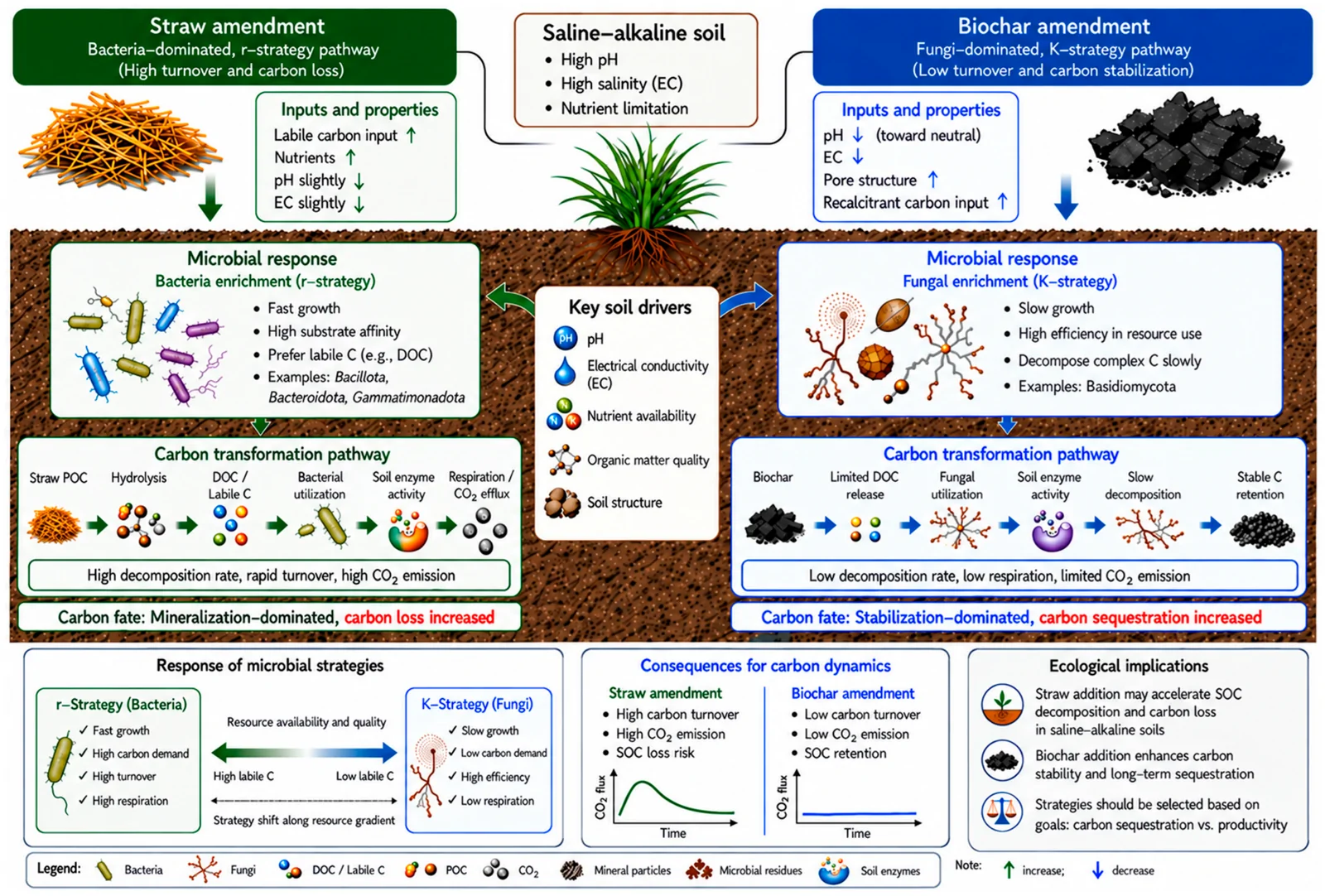
Conceptual framework of contrasting microbial processes and carbon turnover pathways under straw and biochar amendments in saline soil.

This POC/MAOC partitioning not only determines the physical protection of carbon but may also shape microbial life-history strategies by altering substrate availability, thereby regulating carbon turnover [9,19]. In agricultural and forest soils, the coupling between POC/MAOC partitioning and microbial *r/K* strategies has been increasingly documented [13,15,20]. Straw input introduces labile carbohydrates that favor *r*-strategist bacteria and enhance hydrolytic enzyme activity, leading to POC enrichment but also to accelerated native SOC mineralization (positive priming) [13,15]. Biochar input, in contrast, provides recalcitrant aromatic carbon and mineral-protective surfaces that favor K-strategist fungi and sustain oxidative enzyme activity, thereby promoting MAOC accumulation via the microbial necromass pathway and often inducing negative priming [13,15,20]. However, in coastal saline soils, salinity stress increases microbial maintenance costs and reduces carbon use efficiency [11,21], potentially altering this coupling fundamentally [22]. Consequently, it remains largely elusive whether the divergent allocation of carbon into POC and MAOC pools following straw and biochar amendments in salt-affected soils further drives a functional divergence between bacterial and fungal trophic strategies, as well as their ecoenzymatic investments (hydrolytic vs. oxidative), and how these intertwined shifts collectively regulate carbon mineralization (Figure 1).

To bridge these critical knowledge gaps, we established a microcosm experiment using coastal saline soil from the Yellow River Delta amended with homologous reed straw or its derived biochar across three application rates. We systematically evaluated soil salinity indicators, POC and MAOC pools, microbial trophic tendencies, five key carbon and nitrogen cycling extracellular enzymes, and dynamic CO_2_ effluxes. The primary objectives of this study were to: (1) determine how straw and biochar redirect exogenous C allocation between unprotected POC and MAOC components; (2) characterize the divergent shifts in bacterial and fungal life-history strategies (*r* vs. K selection proxies) and their associated hydrolytic versus oxidative ecoenzymatic investments; and (3) unravel the material-specific biological cascades linking microbial life-history adaptations and ecoenzymes to dynamic C mineralization.

## 2. Materials and Methods

### 2.1. Soil Sampling and Preparation of Homologous Amendments

The experimental soil was collected from a representative saline cropland at the Yellow River Delta Saline-Alkali Agro-Ecosystem Observation and Research Station, Shandong Province, China (37°32′ N, 118°39′ E). The region has a warm-temperate semi-humid continental monsoon climate, with cold and dry winters and warm and humid summers. According to the original site records of this study, the mean annual temperature is approximately 12.7 °C, the mean annual precipitation is approximately 564.8 mm, and the mean annual ground temperature is approximately 14.7 °C. The tested soil is a coastal saline fluvo-aquic soil (USDA Soil Taxonomy) characterized by alkaline pH, elevated salinity, poor physical structure and low organic matter content. Its particle-size composition consists of 66.31% silt, 8.45% clay and 25.24% sand. The initial physicochemical properties of the soil were as follows: electrical conductivity (EC), 698.1 μS cm^−1^; pH, 8.0; soil organic matter (SOM), 11.2 g kg^−1^; total nitrogen (TN), 0.44 g kg^−1^; Olsen phosphorus (P), 30.72 mg kg^−1^; available potassium (K), 107.22 mg kg^−1^; soil microbial biomass carbon (SMBC), 0.08 g kg^−1^ (provided by the Yellow River Delta Saline-Alkali Agro-Ecosystem Observation and Research Station).

On 6 May 2025, topsoil samples (0–20 cm plough layer) were collected from the field site using a five-point sampling scheme across five randomly established replicate quadrats (100 cm × 100 cm) and then composited within the same plot. This layer was selected because it is the main zone for organic amendment incorporation, microbial activity, extracellular enzyme production and soil CO_2_ exchange. Visible roots, plant residues, stones and soil fauna were removed manually. The collected soil was homogenized, transported to the laboratory, and divided into subsamples for physicochemical analyses, enzyme assays and indoor pot incubation experiments.

Common reed (*Phragmites australis*) straw was harvested from the dominant natural wetland vegetation in the Yellow River Delta. The straw was air-dried, milled, and sieved to pass a 2-mm mesh prior to use. Homologous biochar was produced from the same batch of reed straw via slow pyrolysis under an oxygen-limited atmosphere in a muffle furnace (SX2-4-10, Shanghai Yiheng Scientific Instrument Co., Ltd., Shanghai, China). The pyrolysis process was conducted at a peak temperature of 500 °C with a heating rate of 10 °C min^−1^ and an isothermal holding time (residence time) of 2 h. For both reed straw and its derived biochar, total C and total N concentrations were determined using an elemental analyzer (Vario EL III, Elementar, Analysensysteme GmbH, Hanau, Germany), and the C/N mass ratio was calculated accordingly. Ash content was measured gravimetrically by combustion in a muffle furnace at 750 °C for 6 h. Material pH and electrical conductivity (EC) were measured in a 1:20 (*w*/*v*) solid-to-deionized water suspension after shaking for 1.5 h. The basic physicochemical properties of the reed straw and its derived biochar are presented in Table 1.

### 2.2. Microcosm Experimental Design and Incubation Management

A 120-day microcosm experiment was conducted with seven treatments: an unamended control (CK); three straw treatments receiving reed straw at 1%, 5%, and 9% of dry soil mass (JF1, JF5, and JF9, respectively); and three biochar treatments receiving biochar derived from the same reed feedstock at the corresponding application rates (TF1, TF5, and TF9, respectively). These rates corresponded to 10, 50, and 90 g of amendment per kilogram of dry soil. The same mass-based rates have been used in a maize straw amendment study [23], while comparable rates of 1%, 5%, and 10% have been examined in a biochar incubation study [24]. At each application rate, equal masses of straw and biochar were added per unit dry soil mass, without adjustment for their respective carbon concentrations. The treatments were therefore compared on an equal-mass rather than an equal-carbon basis. Based on material C contents (Table 1), applying 10, 50, and 90 g kg^−1^ of amendment supplied 4.27, 21.33, and 38.39 g C kg^−1^ dry soil in the straw treatments (JF1, JF5, and JF9) versus 6.12, 30.62, and 55.12 g C kg^−1^ dry soil in the biochar treatments (TF1, TF5, and TF9), respectively.

**Figure 2:**
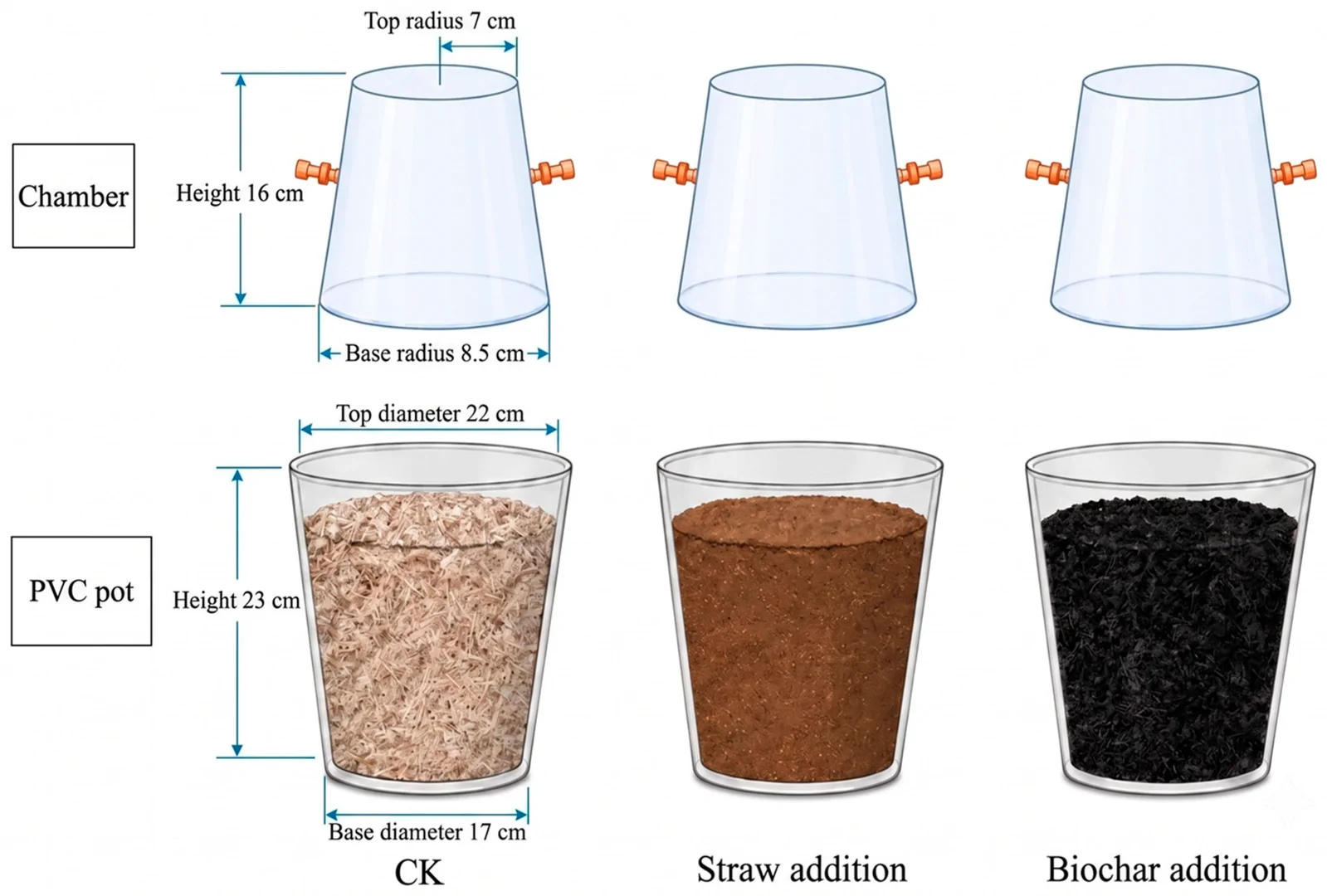
Schematic diagram of the experimental setup for soil incubation and CO_2_ emission measurement.

Each pot was filled with 5.5 kg of dry soil that had been passed through a 2-mm sieve. The soil was mixed thoroughly with the corresponding organic amendment at 55 g (1%), 275 g (5%), or 495 g (9%), and the mixture was then packed into a cylindrical polyvinyl chloride (PVC) pot (height 23 cm, top inner diameter 22 cm, bottom inner diameter 17 cm) (Figure 2). Soil moisture was brought to 60% of the maximum water holding capacity (WHC) with deionized water. The pots were pre-incubated at approximately 20 °C for 15 days to stabilize microbial activity. During incubation, moisture was kept at 60% WHC by weighing the pots and adding deionized water at regular intervals to replace evaporated water. Each treatment had five replicates, giving a total of 35 pots in a completely randomized design.

### 2.3. Soil CO_2_ Efflux Monitoring

Between 09:00 and 11:00 on each measurement day, a static chamber was hermetically mounted onto each PVC pot. Chamber headspace CO_2_ concentrations were monitored continuously over a 1-h interval using an LI-850 portable CO_2_/H_2_O gas analyzer (LI-COR Biosciences, Lincoln, NE, USA). Concurrently, soil temperature and volumetric water content at a depth of 5 cm were recorded. Gas sampling was conducted at 5-day intervals throughout the incubation. General principles and limitations of chamber-based soil respiration measurements are described by Pumpanen et al. [25].

(1) The soil CO_2_ emission rate (μg CO_2_-C·h^−1^) was calculated according to the following equation [26]:

(1) $$F=\frac{M\left(C_{2}-C_{1}\right)}{22.4}\times V_{\mathrm{chamber}}\times w$$

where *F* is the soil CO_2_ emission rate (μg CO_2_-C·h^−1^); *M* is the molar mass of CO_2_ (44.01 g mol^−1^); *C*_1_ and *C*_2_ are the initial and final volumetric fractions (μL L^−1^) of CO_2_ measured within the chamber headspace during the 1 h deployment; 22.4 is the molar volume of an ideal gas at standard temperature and pressure (22.4 L mol^−1^); *V*_chamber_ is the effective internal headspace volume of the chamber (m^3^); *w* is the mass proportion of carbon in CO_2_ (w = 12/44).

(2) Cumulative soil carbon mineralization *Q* (μg CO_2_-C) over the entire incubation was calculated as follows [26]:

(2) $$Q=\sum_{i=1}^{n}24\times F_{i}$$

where *F_i_* is the CO_2_ emission rate on sampling day *i*, and *n* represents the total duration of the incubation in days.

### 2.4. Soil Physicochemical Properties, Carbon Fractions, and Microbial Biomass

Soil bulk density (BD) was determined using the cutting-ring method [27]. Volumetric soil water content was measured using a Theta Probe ML2X connected to an HH2 moisture meter (Delta-T Devices, Cambridge, UK). Soil pH and electrical conductivity (EC) were measured in aqueous soil suspensions using calibrated meters. SOC was determined by potassium dichromate (K_2_Cr_2_O_7_) oxidation with external heating [27]. SOM was estimated by multiplying SOC by 1.724 [28] and was not measured independently. Total nitrogen (TN) was determined by Kjeldahl digestion [27]. Available phosphorus was extracted with 0.5 M NaHCO_3_ and determined colorimetrically using the Olsen method [29]. Available potassium was extracted with neutral 1.0 M ammonium acetate (NH_4_OAc) and quantified by flame photometry [30]. Soil ammonium nitrogen (NH_4_^+^-N) was extracted with 2.0 M KCl and determined colorimetrically using the indophenol blue method [31]. Soil microbial biomass carbon (SMBC) and nitrogen (SMBN) were determined by chloroform fumigation followed by extraction [32].

Soil carbon fractions were determined by particle size fractionation. Briefly, 10.0 g of air-dried soil was dispersed in 30 mL of sodium hexametaphosphate solution (5 g L^−1^) by shaking on a reciprocal shaker at 90 rpm for 18 h. The suspension was passed through a 53 μm sieve and rinsed thoroughly with deionized water. Material retained on the sieve (>53 μm) was oven-dried at 60 °C, weighed, and finely ground for POC determination by dichromate oxidation. MAOC was calculated by subtracting POC from the total SOC [29]. This estimate was based on particle size (<53 μm) and did not directly characterize carbon origin or mineral binding, as it may encompass mineral-bound carbon, fine amendment particles (<53 μm), and soluble carbon.

Extracellular enzyme activities were determined using microplate-based fluorometric and colorimetric assays. The hydrolytic enzymes β-D-glucosidase (BG), β-D-xylosidase (XYL), cellobiohydrolase (CBH), and N-acetyl-β-D-glucosaminidase (NAG) were quantified fluorometrically using their corresponding 4-methylumbelliferone (MUB)-linked substrates (Sigma-Aldrich, St. Louis, MO, USA), following Saiya-Cork et al. [33] and subsequent modifications [34]. For each soil sample, 1 g of fresh soil was suspended in 125 mL of 50 mM buffer and homogenized for 2 h on a constant-temperature shaker. The suspension was continuously mixed during microplate preparation to minimize particle settling. For each assay, 200 μL of soil suspension was transferred to a 96-well microplate. Sample wells received 50 μL of the corresponding 200 μM MUB-linked substrate solution, whereas soil-background blanks contained 200 μL of soil suspension and 50 μL of buffer. Quench controls consisted of 200 μL of soil suspension and 50 μL of a 10 μM MUB standard (Molecular Devices, Sunnyvale, CA, USA), while substrate controls contained 200 μL of buffer and 50 μL of substrate solution. Reference-standard wells contained 200 μL of buffer and 50 μL of the 10 μM MUB standard. The prepared microplates were incubated in the dark at 25 °C for 4 h, after which 50 μL of 0.5 M NaOH was added to terminate the reaction. Fluorescence was measured with a SpectraMax Gemini microplate reader (Molecular Devices, Sunnyvale CA, USA) at excitation and emission wavelengths of 365 and 450 nm, respectively. Fluorometric enzyme activities were corrected for background fluorescence, substrate controls, and soil-matrix quenching using the MUB reference standard. Polyphenol oxidase (PPO) activity was determined colorimetrically using L-3,4-dihydroxyphenylalanine (L-DOPA) (Sigma-Aldrich, St. Louis, MO, USA) as the substrate, following the microplate assay procedure described by Saiya-Cork et al. [33] and German et al. [34] with minor modifications. Soil suspension and L-DOPA solution were incubated in the dark at 25 °C, and PPO activity was quantified from the increase in absorbance at 450 nm after correction for soil-background and substrate controls. Enzyme activities were expressed on an oven-dry soil basis. The units for each enzyme are as follows: PPO, mg d^−1^ g^−1^; CBH, nmol h^−1^ g^−1^; XYL, μmol h^−1^ g^−1^; BG and NAG, μmol d^−1^ g^−1^.

For microbial community analysis, soil genomic DNA was extracted using a FastDNA SPIN Kit (MP Biochemicals, Solon, OH, USA). The V4 region of the bacterial 16S rRNA gene was amplified using the 515F/806R primer pair [35], whereas the fungal ITS1 region was amplified using the ITS5-1737F/ITS2-2043R primer pair. The ITS5/ITS2 primer framework originates from White et al. [36]. Amplicon libraries were sequenced using an Illumina HiSeq 2500 platform (Illumina, San Diego, CA, USA). Raw sequences were processed using QIIME [37], chimeras were screened using UCHIME [38], and high-quality sequences were clustered into operational taxonomic units at 97% sequence similarity using UPARSE [39]. Taxonomic assignments were obtained using a BLAST-based approach [40]. The sequence data of the fungi and bacteria were deposited with the NCBI (PRJNA1531304).

Microbial trophic tendencies were explored using phylum-level relative-abundance ratios, following the general framework applied by Li et al. [41] and Li et al. [42]. The bacterial ratio was calculated as ((Acidobacteriota + Actinomycetota + Chloroflexota)/(Bacillota + Gemmatimonadota + Bacteroidota)), and the fungal ratio as Basidiomycota/Ascomycota. Under the classification adopted here, higher ratios indicated a greater relative contribution of taxa designated as putative oligotrophs, whereas lower ratios indicated a greater relative contribution of putative copiotrophs. Because indices based on the relative abundance of entire taxonomic phyla do not directly measure physiological life-history traits, these ratios were used to reflect shifts in microbial community composition and the relative abundance of potential oligotrophs and copiotrophs.

### 2.5. Statistical Analyses

All data were reported as means ± standard deviation (SD). Differences in end-point soil physicochemical parameters, SOC fractions, microbial biomass, extracellular enzyme activities, bacterial and fungal life-history proxies, and soil CO_2_ emission rates among the seven treatments were examined using one-way analysis of variance (ANOVA). Where the assumption of homogeneity of variance was satisfied, post hoc pairwise comparisons were performed using Duncan’s multiple range test at a significance threshold of *p* < 0.05.

Associations between microbial community dissimilarities and environmental distances were evaluated using Mantel tests [43], implemented in the vegan package (v2.7-5) in R (v4.3.0) (R Foundation for Statistical Computing, Vienna, Austria). Microbial community dissimilarities were calculated using the Bray–Curtis index [44], whereas Euclidean distance matrices were constructed for soil environmental variables. The significance of Mantel’s r was assessed using 4999 permutations. Partial least squares structural equation modeling (PLS-SEM) was used to assess hypothesized relationships among soil variables, microbial indicators, enzyme activities, and CO_2_ emissions within the straw and biochar datasets, following reporting principles described by Hair et al. [45]. Models were fitted using SmartPLS (v4.1.1.8; SmartPLS GmbH, Bönningstedt, Germany). Continuous variables were standardized using Z-score transformations. Pathways were specified based on ecological hypotheses and bivariate correlation patterns, without automated stepwise selection. Non-parametric bootstrapping with 5000 resamples was used to obtain standard errors, t-statistics, *p*-values, and 95% confidence intervals. Standardized path coefficients (β) and coefficients of determination (*R*^2^) were reported. Predictor multicollinearity was assessed using variance inflation factors (VIFs; all values < 5.0). The models were interpreted as conditional associations rather than independent demonstrations of causality. Given the limited sample size (*n* = 12 per model), PLS-SEM was used to explore statistical associations rather than to prove causal pathways.

ChatGPT (OpenAI, GPT-6) was used to assist in preparing Figure 1 and Figure 2 based on the authors’ conceptual framework and experimental design. Both figures were reviewed and edited by the authors to ensure scientific accuracy.

## 3. Results

### 3.1. Effects of Straw and Biochar Amendment on Soil Physicochemical Properties

Both straw and biochar additions significantly altered soil temperature, moisture content, bulk density, and salinity (*p* < 0.05; Table 2). Compared with CK, all amended treatments had significantly lower soil temperatures, decreasing from 26.6 °C to 25.0–25.7 °C, and significantly higher soil moisture contents. Under straw addition, moisture content increased progressively from 21.6% in CK to 24.4%, 28.1%, and 32.0% in JF1, JF5, and JF9, respectively. The corresponding values in TF1, TF5, and TF9 were 27.7%, 27.8%, and 25.1%, respectively; all exceeded CK, but showed no monotonic increase with application rate. Straw and biochar reduced soil bulk density by 9.2–33.1% and 7.7–30.0%, respectively, with greater reductions generally occurring at higher application rates. Straw had a relatively small effect on soil pH, whereas biochar lowered the pH from 8.5 in CK to 7.8, 7.6, and 7.3 in TF1, TF5, and TF9, respectively. Both amendments also significantly reduced the electrical conductivity (EC). EC values in JF9 and TF9 decreased to 415.3 and 409.5 μS cm^−1^, respectively, approximately 40% below the CK value of 698.1 μS cm^−1^.

Under straw addition, TN, Olsen-P, and available K generally increased with application rate, reaching 1.03 g kg^−1^, 70.62 mg kg^−1^, and 425.68 mg kg^−1^, respectively, in JF9 (*p* < 0.05; Table 2). NH_4_^+^–N concentrations ranged from 6.18 to 6.53 mg kg^−1^, consistently exceeding the CK value of 4.92 mg kg^−1^. Biochar produced smaller increases in TN, while Olsen-P and available K were highest in TF5, at 48.36 and 621.83 mg kg^−1^, respectively (*p* < 0.05; Table 2). However, the NH_4_^+^–N concentrations decreased significantly relative to CK, ranging from 1.24 to 1.49 mg kg^−1^ in the biochar treatments. Soil microbial biomass carbon (SMBC) increased markedly under both amendments. As the application rate increased, SMBC rose from 0.37 to 0.68 g kg^−1^ under straw addition and from 0.12 to 0.41 g kg^−1^ under biochar addition, compared with 0.08 g kg^−1^ in CK. Straw generally produced larger increases in SMBC than biochar (*p* < 0.05; Table 2).

### 3.2. SOC Increase and Differential POC and MAOC Enrichment in Response to Straw and Biochar Addition

Mean SOC concentrations were higher in both straw and biochar amended soils than in the unamended control (CK) and increased with application rate (*p* < 0.05; Table 2). SOC concentration was 6.49 g kg^−1^ in CK, compared with 8.64, 22.93, and 34.27 g kg^−1^ in JF1, JF5, and JF9, respectively, and 10.10, 21.73, and 29.97 g kg^−1^ in TF1, TF5, and TF9, respectively. At matched application rates, mean SOC was higher under biochar at 1% and under straw at 9%, whereas the means were similar in magnitude at 5%.

**Figure 3:**
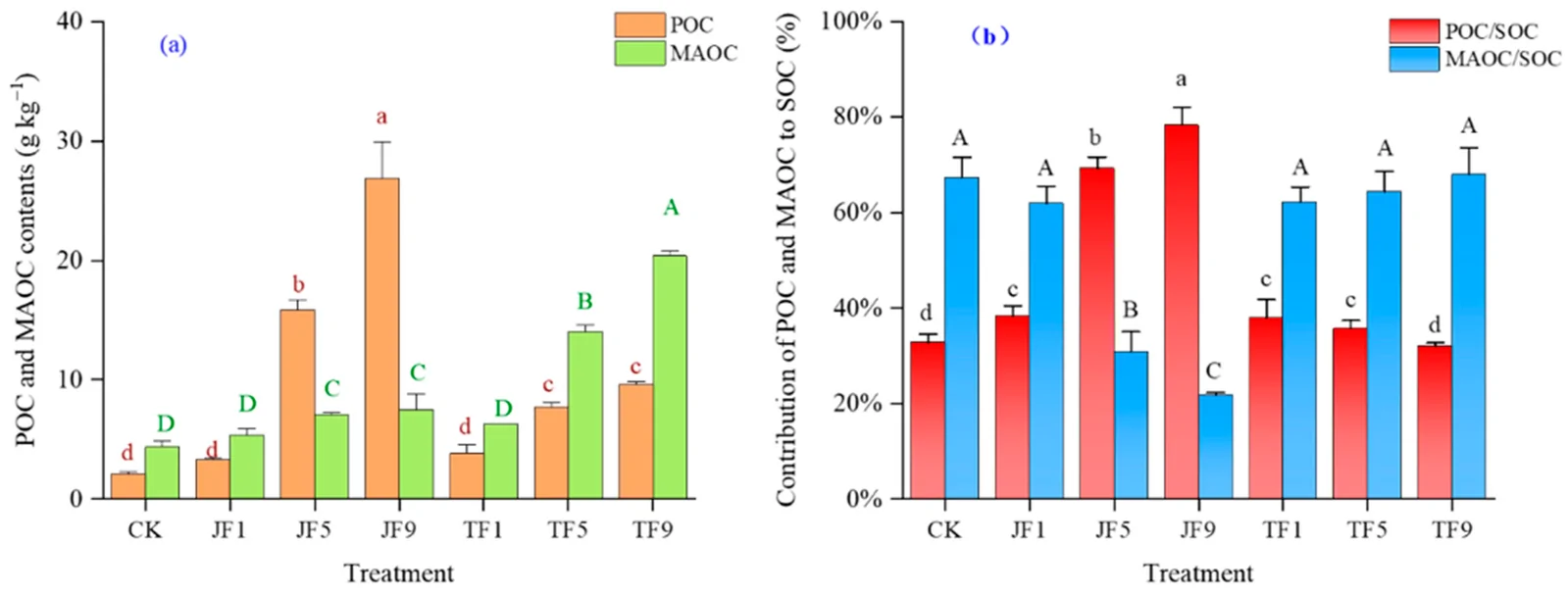
Effects of straw and biochar amendments on soil particulate organic carbon (POC) and mineral-associated organic carbon (MAOC): (**a**) concentrations; (**b**) contributions to total SOC. Error bars indicate standard deviations. Different lowercase letters for POC and uppercase letters for MAOC indicate significant differences among treatments (*p* < 0.05). Treatments: CK, unamended control; JF1, JF5, and JF9, reed straw applied at 1%, 5%, and 9% of dry soil mass, respectively; TF1, TF5, and TF9, homologous reed biochar applied at 1%, 5%, and 9% of dry soil mass, respectively.

Both straw and biochar significantly increased the POC and MAOC concentrations relative to CK (*p* < 0.05; Figure 3), although their effects on SOC composition differed. In JF1, JF5, and JF9, the POC concentrations increased by 55.7%, 647.6%, and 1168.9%, respectively, whereas the corresponding increases in MAOC were 22.2%, 62.0%, and 71.2%. With increasing straw application rate, the contribution of POC to SOC rose from 32.7% in CK to 38.2%, 69.1%, and 78.2% in JF1, JF5, and JF9, respectively. Conversely, the MAOC contribution declined from 67.3% to 61.8%, 30.9%, and 21.7%, respectively. These changes reflected a progressively greater contribution of POC to SOC despite concurrent increases in MAOC concentration. In biochar amended soils, POC and MAOC concentrations increased by 80.7–352.8% and 43.7–366.1%, respectively, relative to CK (*p* < 0.05; Figure 3). Their proportional contributions to SOC, however, remained within 32.0–37.9% for POC and 62.1–68.0% for MAOC. Thus, MAOC remained the dominant SOC fraction under biochar addition, whereas increasing the straw application rates shifted SOC composition toward a greater contribution of POC.

### 3.3. Temporal Dynamics of Soil CO_2_ Emissions Under Straw and Biochar Addition

**Figure 4:**
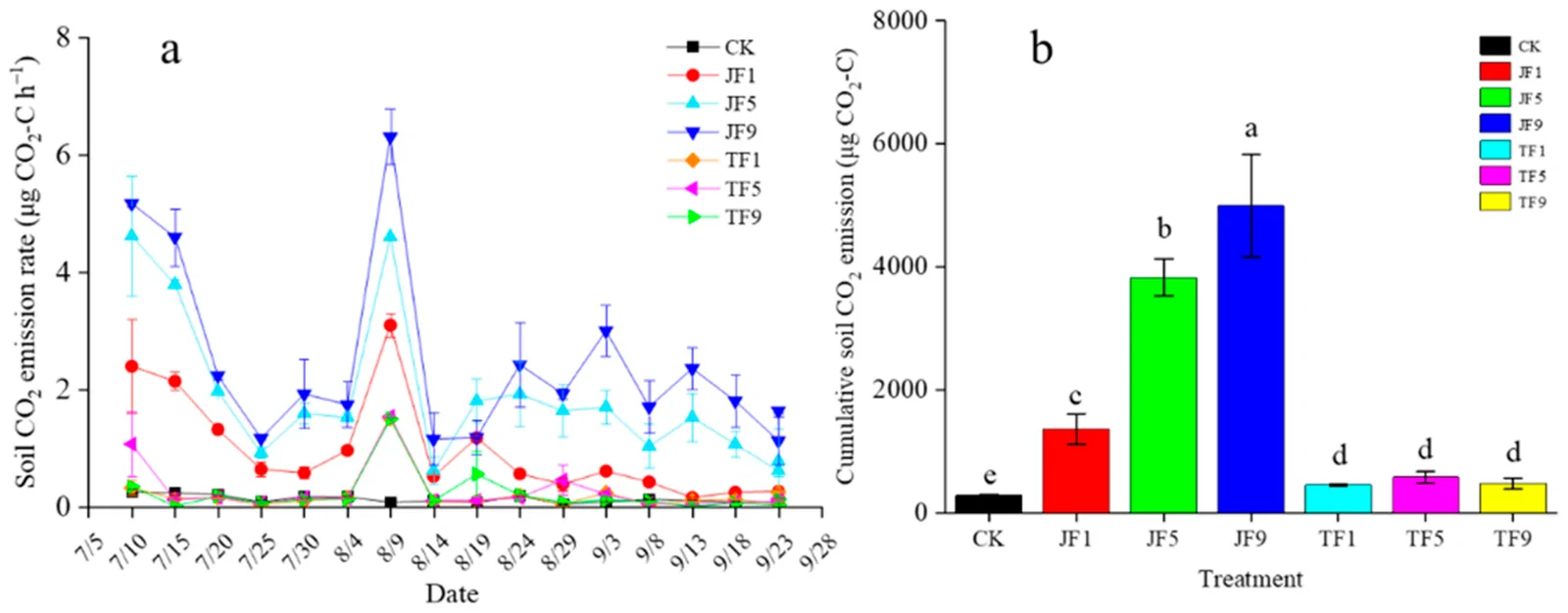
Dynamics of soil CO_2_ emissions under straw and biochar amendments: (**a**) emission rates over the measurement period; (**b**) cumulative CO_2_–C release. Error bars indicate standard deviations. Different lowercase letters in panel (**b**) indicate significant differences among treatments (*p* < 0.05). Treatments: CK, unamended control; JF1, JF5, and JF9, reed straw applied at 1%, 5%, and 9% of dry soil mass, respectively; TF1, TF5, and TF9, homologous reed biochar applied at 1%, 5%, and 9% of dry soil mass, respectively.

Soil CO_2_ emissions fluctuated over time rather than declining monotonically throughout the observation period (*p* < 0.05; Figure 4a). Higher release rates under straw were observed mainly at the medium and high application rates, with JF5 and JF9 exceeding JF1 on several measurement dates. Rates in the three biochar treatments were generally lower than those in the straw treatments, and differences among application rates were less pronounced under biochar (*p* < 0.05; Figure 4a).

Cumulative soil CO_2_ emissions increased with straw application rate (*p* < 0.05; Figure 4b). All three biochar treatments had higher mean cumulative release than CK but lower means than the straw treatments (*p* < 0.05; Figure 4b). Although biochar introduced 43.6% more C than straw at matched mass rates, the net respiratory increase above CK normalized per unit of added C was 7.5–19.6 times higher under straw than under biochar. Thus, the increases in SOC concentration under both amendments were accompanied by different degrees of respiratory enhancement. It should be noted that these comparisons refer to total CO_2_ release from the soil–amendment system within the observation period relative to CK, and do not directly indicate the proportion of added amendment C mineralized.

### 3.4. Responses of Microbial Community Composition and Potential Extracellular Enzyme Activities to Straw and Biochar Addition

**Figure 5:**
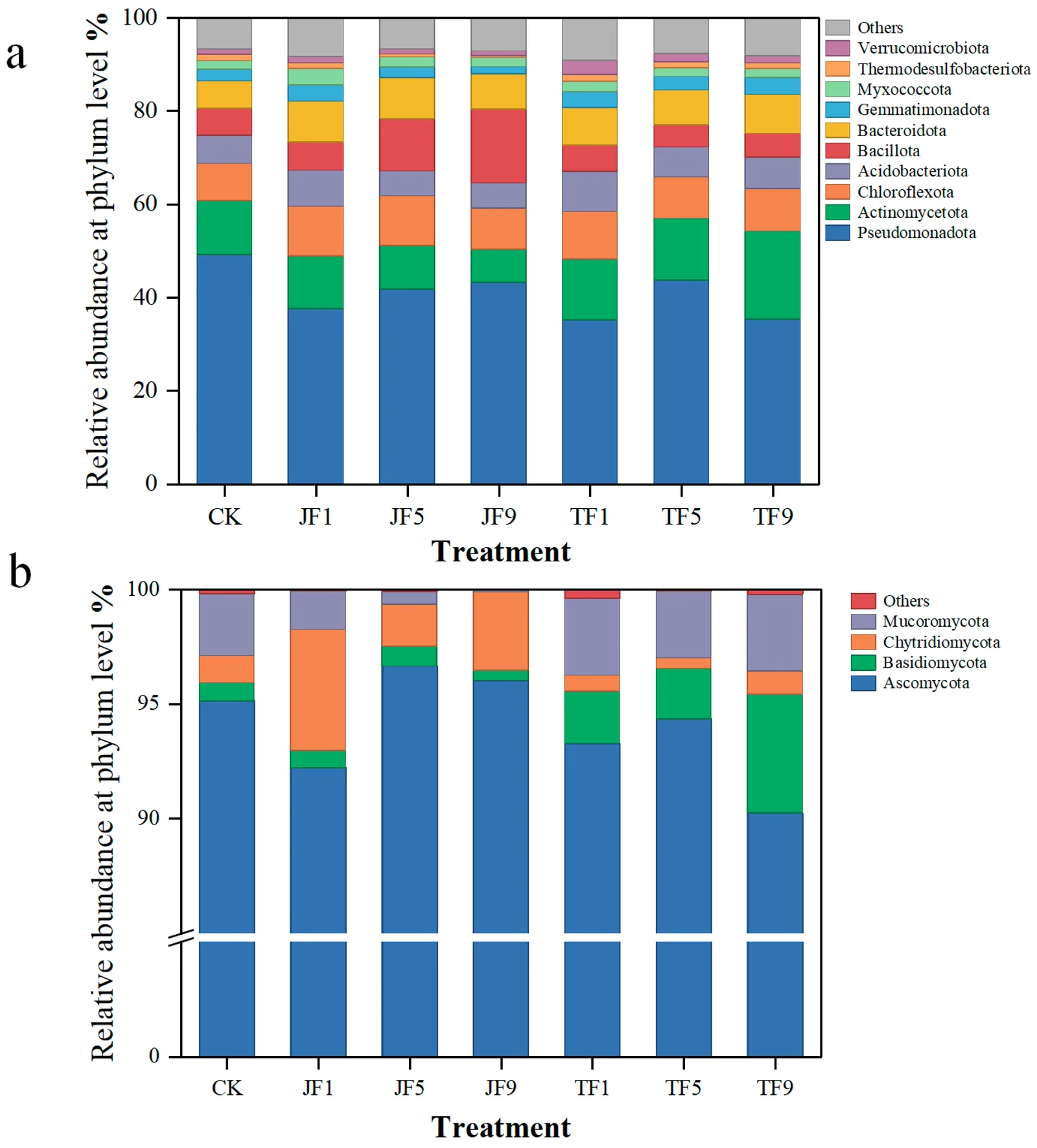
Taxonomic composition and relative abundance of soil (**a**) bacterial and (**b**) fungal communities at the phylum level under straw and biochar amendments. Treatments: CK, unamended control; JF1, JF5, and JF9, reed straw applied at 1%, 5%, and 9% of dry soil mass, respectively; TF1, TF5, and TF9, homologous reed biochar applied at 1%, 5%, and 9% of dry soil mass, respectively.

Although the dominant bacterial phyla remained consistent across all treatments, straw and biochar amendments distinctly reshaped the relative abundances of specific taxa, particularly those associated with microbial life-history strategies (Figure 5). At the phylum level, bacterial communities across all treatments were dominated by Pseudomonadota (Proteobacteria), Actinomycetota, Chloroflexota, Acidobacteriota, Bacillota, Bacteroidota, and Gemmatimonadota (Figure 5a). The mean relative abundance of Pseudomonadota ranged from 35.2% to 49.3%, with no consistent trend across amendment application rates (*p* < 0.05; Figure 5a). In contrast, Actinomycetota and Bacillota exhibited opposing responses to increasing straw addition. The mean relative abundance of Actinomycetota progressively decreased from 11.7% in CK to 7.0% in JF9, whereas that of Bacillota increased from 5.7% to 16.0%. Biochar addition produced a different pattern: the relative abundance of Actinomycetota reached 18.9% at the highest application rate (TF9), while Bacillota remained comparatively low across the biochar treatments (*p* < 0.05; Figure 5a).

Fungal communities were overwhelmingly dominated by Ascomycota across all treatments, with mean relative abundances ranging from 90.3% to 96.7% (*p* < 0.05; Figure 5b). Straw addition had little effect on the relative abundance of Basidiomycota, which ranged from 0.43% to 0.87% and declined to 0.43% in JF9. In comparison, biochar addition increased the relative abundance of Basidiomycota to 2.27%, 2.20%, and 5.20% in TF1, TF5, and TF9, respectively, with the largest increase observed in TF9 (*p* < 0.05; Figure 5b). Among the less abundant fungal phyla, the relative abundance of Chytridiomycota increased to 5.30% in JF1, whereas that of Mucoromycota decreased to approximately 0.10% in JF9.

**Figure 6:**
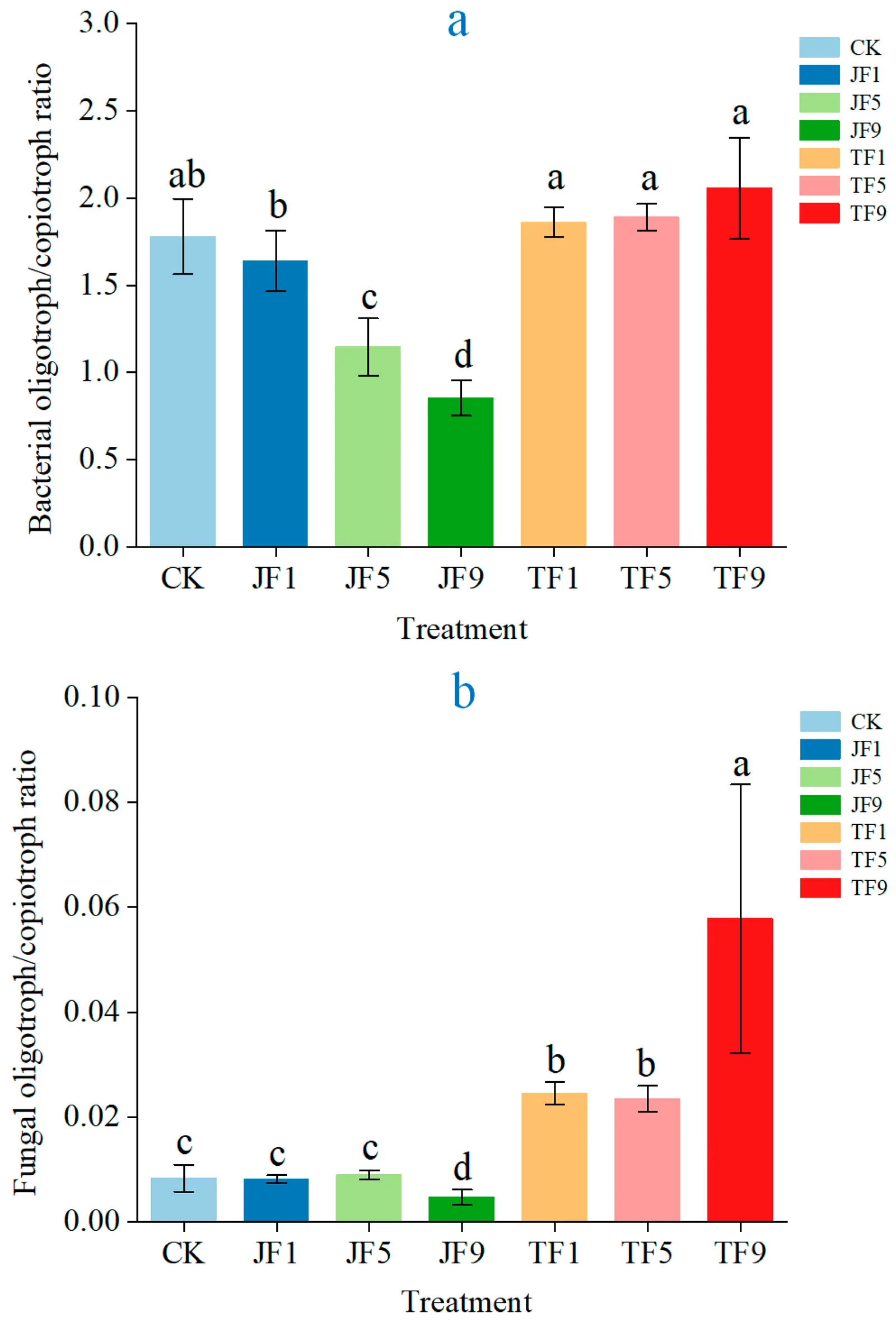
Phylum-level microbial trophic proxies under straw and biochar amendments: (**a**) bacterial and (**b**) fungal oligotroph/copiotroph ratios. Ratios were calculated as described in Section 2.4. Error bars indicate standard deviations. Different lowercase letters indicate significant differences among treatments (*p* < 0.05). Treatments: CK, unamended control; JF1, JF5, and JF9, reed straw applied at 1%, 5%, and 9% of dry soil mass, respectively; TF1, TF5, and TF9, homologous reed biochar applied at 1%, 5%, and 9% of dry soil mass, respectively.

Furthermore, the bacterial oligotroph to copiotroph ratio was 1.82 in CK and decreased progressively with increasing straw application rate, reaching 1.65, 1.14, and 0.86 in JF1, JF5, and JF9, respectively (Figure 6). These values represented reductions of approximately 9.4%, 37.2%, and 52.4% relative to CK. Based on this taxonomic proxy, the pattern suggested an increasingly pronounced shift toward potential copiotrophic taxa with increasing straw addition. In contrast, the ratio reached 1.86, 1.89, and 2.06 in TF1, TF5, and TF9, respectively, exceeding the CK value in all biochar treatments (Figure 6). These higher ratios were consistent with a shift toward potential oligotrophic taxa, with the strongest tendency observed in TF9.

Straw and biochar elicited contrasting responses in potential extracellular enzyme activities across application rates (*p* < 0.05; Table 3). Under straw addition, PPO, CBH, XYL, BG, and NAG activities generally increased with increasing application rate. In JF9, CBH, XYL, BG, and NAG activities were 7.8, 9.1, 4.8, and 4.3 times those in CK, respectively (*p* < 0.05; Table 3). Biochar addition slightly increased the PPO activity, whereas CBH and NAG activities declined progressively with increasing application rate, and XYL and BG activities remained comparatively low (Table 3). These patterns indicate that fresh straw enhanced the potential for the extracellular enzymatic degradation of structural carbon compounds and nitrogen-containing substrates, whereas biochar did not induce a comparable hydrolytic enzyme response (Table 3).

### 3.5. Environmental and Microbial Associations with Soil Carbon Fractions and Soil CO_2_ Emissions Under Straw and Biochar Addition

**Figure 7:**
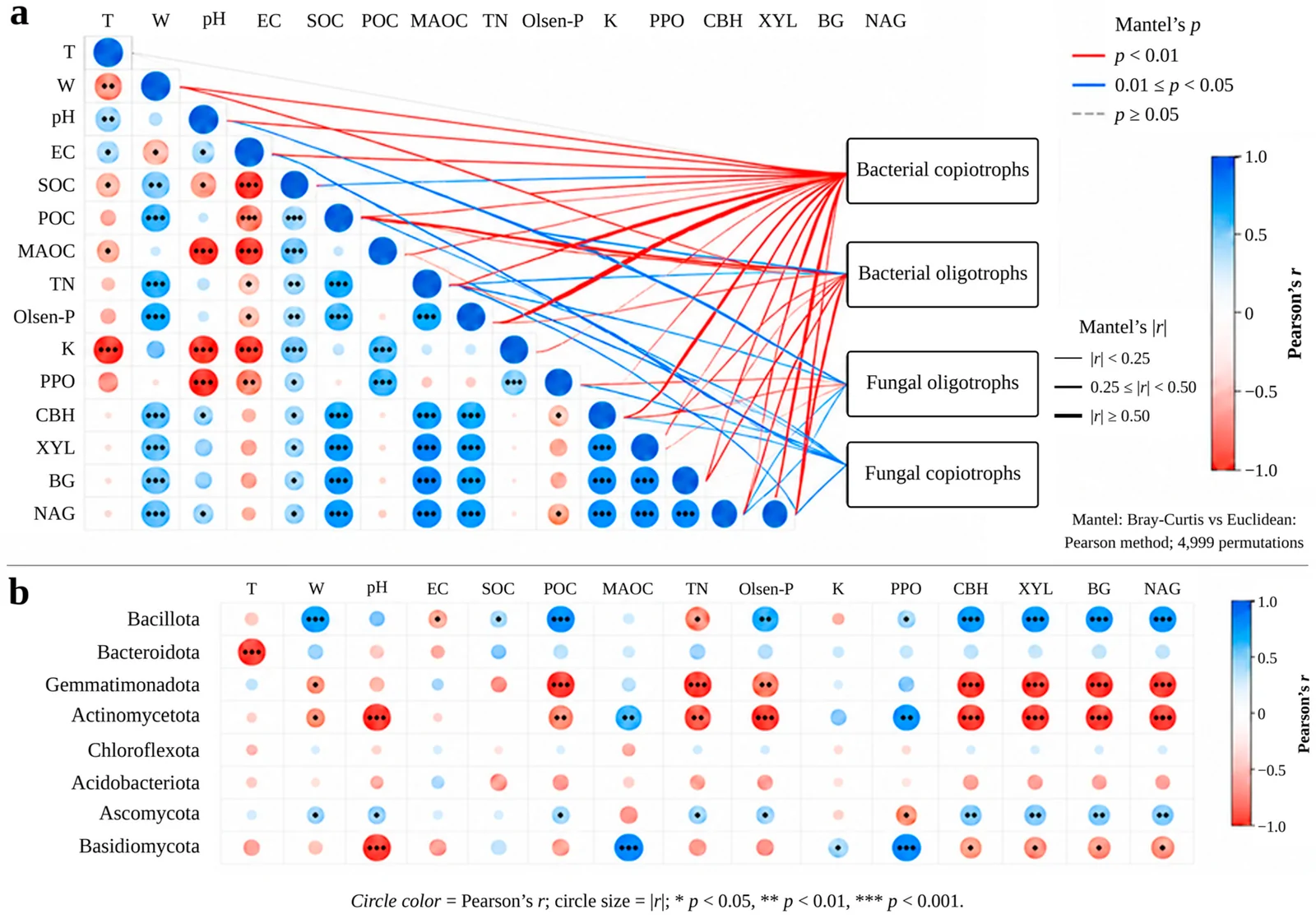
Pearson correlations and Mantel associations among soil properties, carbon fractions, enzymes, and microbial groups. (**a**) Environmental correlations with Mantel networks for four microbial trophic groups; (**b**) dominant phyla versus environmental variables. Circle color indicates Pearson’s r (blue, positive; red, negative), and size indicates |r|. Asterisks denote * *p* < 0.05, ** *p* < 0.01, and *** *p* < 0.001. Mantel line colors indicate significance (red, *p* < 0.01; blue, 0.01 ≤ *p* < 0.05; dashed gray, *p* ≥ 0.05); widths indicate |r| (<0.25, 0.25–0.50, and ≥0.50). T, temperature; W, moisture. Other abbreviations are defined in Table 1 and Table 2 and Figure 3.

Pearson correlation analysis revealed coordinated variation among soil carbon fractions, nutrient concentrations, and potential extracellular enzyme activities (*p* < 0.05; Figure 7a). POC was positively correlated with TN and the activities of CBH, XYL, BG, and NAG, whereas EC showed negative correlations with SOC, POC, and several hydrolytic enzyme activities. The four hydrolytic enzymes also exhibited positive correlations with one another. At the phylum level, the relative abundance of Bacillota was positively correlated with POC and multiple hydrolytic enzyme activities, whereas Gemmatimonadota showed negative correlations with POC, TN, and hydrolytic enzyme activities (Figure 7b).

Mantel tests further revealed that compositional shifts in bacterial and fungal copiotrophic and oligotrophic taxa were significantly associated with multiple parameters, encompassing soil moisture, pH, EC, SOC fractions, nutrients, and extracellular enzyme activities (Figure 7a). However, the specific environmental association patterns varied considerably among the different microbial groups. The significant Mantel associations were distributed across two statistical thresholds (*p* < 0.01 and 0.01 ≤ *p* < 0.05), with several correlations yielding Mantel |r| values of 0.25–0.50 or ≥ 0.50 (Figure 7). These results underscore the presence of robust, yet varying degrees of covariation between the assembly of microbial life-history strategy taxa and soil resource/environmental gradients (Figure 7). Importantly, these significant correlations were not exclusively driven by any single environmental variable. Instead, this multi-factor linkage suggests that the responses of bacterial and fungal communities in saline soils are governed by the comprehensive interplay of water–salt regimes, carbon pools, and nutrient availability.

**Figure 8:**
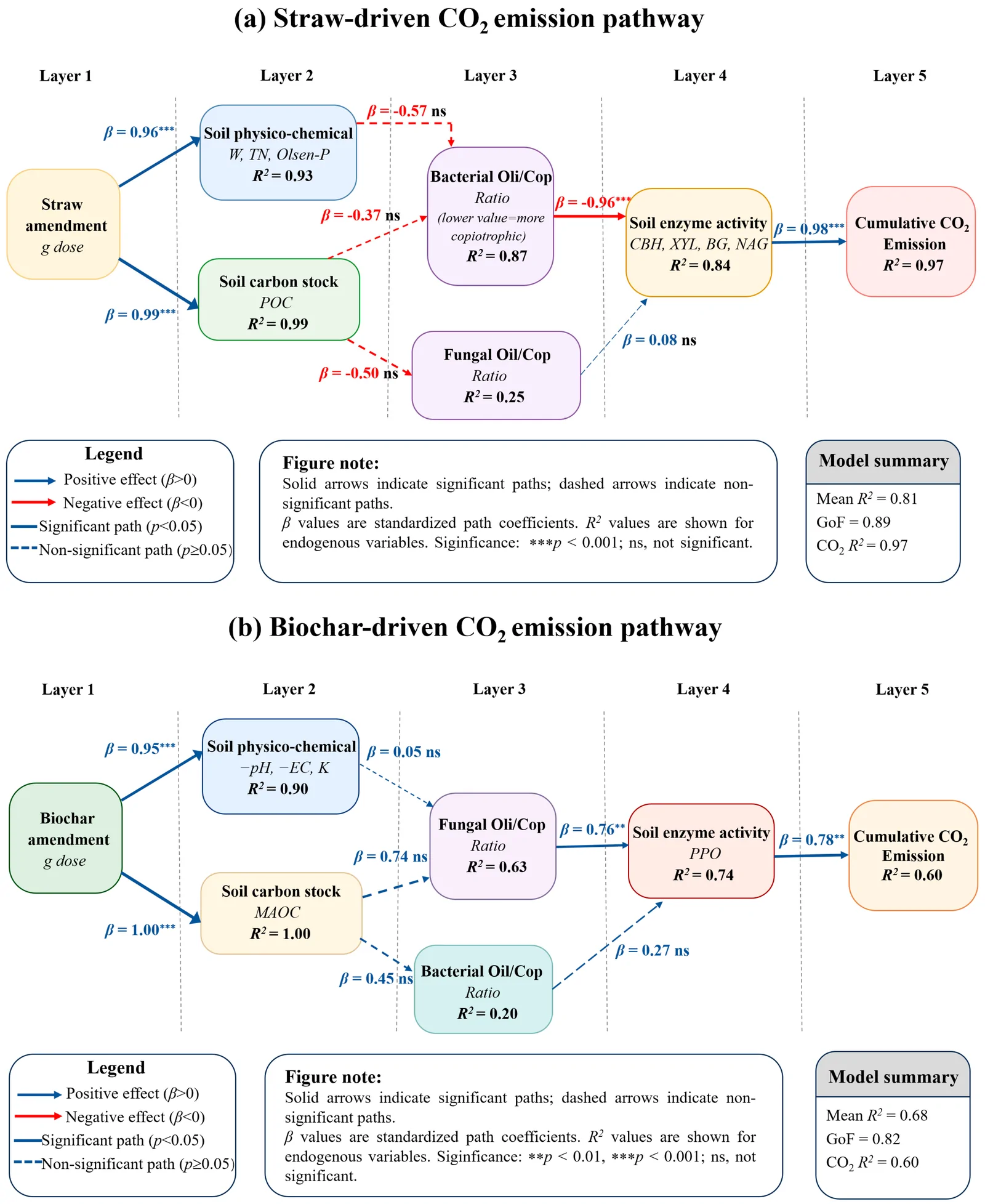
Partial least squares structural equation models for (**a**) straw and (**b**) biochar amendments (*n* = 12 per model). Arrows indicate hypothesized relationships; solid and dashed arrows indicate significant and non-significant paths, respectively. Blue and red denote positive and negative coefficients. Values are standardized path coefficients (β; ** *p* < 0.01, *** *p* < 0.001; ns, not significant), evaluated with 5000 bootstrap resamples. *R*^2^ indicates explained variance. Average *R*^2^ and goodness of fit (GoF) were 0.81 and 0.89 for straw and 0.68 and 0.82 for biochar, respectively.

In the straw model, amendment rate was positively associated with the environmental composite comprising soil moisture, TN, and Olsen-P (β = 0.96, *p* < 0.001) and with POC (β = 0.99, *p* < 0.001). The bacterial taxonomic composition ratio was negatively associated with the hydrolytic enzyme composite comprising CBH, XYL, BG, and NAG (β = −0.96, *p* < 0.001), which was positively associated with cumulative CO_2_ release (β = 0.98, *p* < 0.001). However, the path from the environmental composite to the bacterial taxonomic composition ratio and the paths from POC to either the bacterial or fungal taxonomic composition ratio were not statistically significant. The path from the fungal taxonomic composition ratio to the hydrolytic enzyme composite was also not significant. The model explained 84% of the variance in the hydrolytic enzyme composite and 97% of the variance in cumulative CO_2_ emissions (Figure 8a).

In the biochar model, amendment rate was positively associated with the environmental composite comprising −pH, −EC, and available K (β = 0.95, *p* < 0.001) and with MAOC (Figure 8b) (β = 1.00, *p* < 0.001). The fungal taxonomic composition ratio was positively associated with PPO activity (β = 0.76, *p* < 0.01), and PPO activity was positively associated with cumulative CO_2_ emissions (β = 0.78, *p* < 0.01). The paths from the environmental composite to the fungal taxonomic composition ratio, from MAOC to either the bacterial or fungal taxonomic composition ratio, and from the bacterial taxonomic composition ratio to PPO were not statistically significant. The model explained 74% of the variance in PPO activity and 60% of the variance in cumulative CO_2_ emissions (Figure 8b).

## 4. Discussion

### 4.1. Straw and Biochar Drive Divergent Carbon Allocation Between POC and MAOC Pools

When evaluating the carbon sequestration potential of degraded soils, the physical and chemical fate of newly introduced carbon is often more decisive than the apparent increase in total carbon [9]. The study reveals that homologous straw and biochar derived from the same reed feedstock elicited fundamentally divergent carbon allocation trajectories in coastal saline soils: the addition of fresh straw increased the contribution of POC to total SOC to 78.2%, whereas homologous biochar maintained the absolute quantitative dominance of MAOC across all application gradients (62.1–68.0%) (Figure 3). Although equal-mass application introduced 43.6% more C under biochar than under straw (Table 1), using relative proportions (POC/SOC and MAOC/SOC) eliminates the effect of unequal initial C inputs. Moreover, even at comparable C inputs—such as JF9 (38.39 g C kg^−1^) versus TF5 (30.62 g C kg^−1^)—straw predominantly enriched POC (78.2% of SOC), whereas biochar maintained MAOC dominance (64.5% of SOC), confirming that carbon partitioning was governed by amendment properties rather than initial C dosage.

The measured baseline properties and responsive soil physicochemical data help explain the potential mechanisms behind this divergence (Table 2). Fresh straw consists primarily of coarse polysaccharide fragments that initially reside as discrete residues (>53 μm). Because the native saline soil possesses an inherently poor physical texture dominated by silt (66.31%) with minimal reactive clay (8.45%), and is constrained by severe osmotic stress (initial EC of 698.1 μS cm^−1^), the limited reactive mineral surfaces may restrict rapid organo-mineral association under a massive residue surge. Consequently, these plant fragments physically fail to associate with minerals, remaining stranded within the vulnerable POC pool [7,10]. Conversely, biochar contains abundant condensed aromatic structures that can physically fragment into fine particles (<53 μm), directly entering the operationally defined MAOC fraction upon physical homogenization. Furthermore, biochar surfaces are enriched with oxygen containing functional groups, and its incorporation systematically buffered the soil pH from 8.5 down to 7.3–7.8 (Table 2). Previous studies suggest that under such alleviated alkaline conditions, oxygen-containing functional groups on biochar surfaces may interact with soil minerals to promote organo-mineral associations [16]; however, direct spectroscopic or density-fractionation evidence would be required to quantify true mineral binding.

In non-saline agricultural soils, fresh straw often converts efficiently into microbial necromass via in vivo turnover to form MAOC [46]. In the studied coastal saline soil, however, straw addition predominantly accumulated within the POC pool during the 120-day incubation. In contrast, pyrolysis into biochar shifted C allocation directly toward the MAOC fraction—likely via the physical introduction of fine biochar particles and potential mineral interactions—thereby bypassing POC accumulation.

### 4.2. Shifts in Microbial Community Composition and Extracellular Enzyme Activities

The chemical heterogeneity of exogenous organic matter not only dictates the physical state of soil carbon but fundamentally remodels the microbial community composition (potential copiotrophs vs. oligotrophs) and ecoenzymatic resource allocations of microbial communities (Figure 1, Figure 5 and Figure 6). Sequencing and enzyme activity data confirmed that straw significantly stimulated the massive proliferation of bacteria (especially Bacillota), pushing the community toward potential copiotrophs while triggering a comprehensive surge in various hydrolytic enzyme activities. Biochar behaved quite differently. It effectively suppressed hydrolytic enzymes, specifically enriched fungal Basidiomycota, shifted the community toward potential oligotrophs, and maintained high polyphenol oxidase (PPO) activity (Figure 5 and Figure 6, Table 3).

This successional direction is primarily controlled by substrate availability (Table 2, Figure 7). Straw brought massive amounts of easily soluble polysaccharides to the barren saline soil, rapidly awakening dormant opportunistic bacteria (potential copiotrophs) [3]. To sustain their rapid proliferation, these bacteria actively secreted hydrolytic enzymes to acquire available nutrients from the matrix [19]. This behavior is quantitatively corroborated by the exponential 4.3- to 9.1-fold increases in CBH, XYL, BG, and NAG activities observed in the JF9 treatment (Table 3). The massive hydrolytic enzyme repertoire reflects an exploitative acquisition strategy dedicated to mining both simple carbohydrates and structural plant polymers [19], driving rapid substrate catabolism at the expense of high metabolic maintenance.

Unlike previous studies confirming that biochar stimulates overall microbial biomass and enzymatic activities in fertile soils by improving soil aeration and physical habitat [47], our study shows that biochar in our coastal saline soil had a marked inhibitory effect on hydrolytic enzymes, particularly CBH and NAG (Table 3). This suppression originates from the absence of bioavailable polysaccharides in biochar and the strong physical sorption and steric hindrance imposed by its porous surfaces on extracellular enzymes and catalytic substrates [17,18]. Furthermore, biochar mitigated osmotic stress by lowering electrical conductivity (~40%) and neutralizing soil alkalinity (pH from 8.5 to 7.3; Table 2). Rather than stimulating opportunistic copiotrophs, this ameliorated habitat provided a competitive advantage to oligotrophic taxa capable of tolerating and degrading polycyclic aromatics [42]. Taxa within Basidiomycota possess specialized oxidative enzyme systems (e.g., PPO and peroxidases) capable of mining complex aromatic matrices under resource-limited conditions [42]. The 12-fold increase in the abundance of the Basidiomycota phylum during biochar treatment, along with the increase in PPO activity, strongly supports this “oxidation economy” model dominated by potential oligotrophic fungi (Figure 5, Figure 6 and Figure 7). This is consistent with the microbial response patterns in our previous study on soil redistribution on the Loess Plateau [42], where we showed that greater chemical stability of soil organic matter, such as a higher aromatic-to-carboxyl carbon ratio, drove bacterial and fungal communities toward K-strategy succession and enhanced polyphenol oxidase secretion for recalcitrant carbon use. Therefore, the chemical recalcitrance of the input material fundamentally determines the soil’s substrate-degrading enzyme profile and microbial evolutionary trajectory.

### 4.3. Microbial and Enzymatic Mechanisms Regulating Divergent Soil CO_2_ Emissions

The amount of carbon that escapes back to the atmosphere via respiration after organic matter supplementation is a critical metric for true carbon sequestration efficiency [14]. Gas monitoring revealed a stark contrast: straw triggered an explosive peak in carbon dioxide emissions, whereas biochar, despite multiplying the total soil carbon content, exhibited remarkably flat and limited respiratory emissions (Figure 4). Normalizing net CO_2_-C release above CK per unit of added C demonstrated a 7.5- to 19.6-fold higher apparent system respiratory response under straw than under biochar, indicating that the contrasting total CO_2_ effluxes were driven primarily by differences in substrate decomposability and potential priming effects rather than initial C inputs. Exploratory PLS-SEM models indicated statistical associations underlying these contrasting respiratory responses (Figure 8). Upon entering the soil, the massive amount of free, coarse particulate carbon from straw directly fueled the frantic expansion of potential copiotrophic bacteria (Figure 1, Figure 7 and Figure 8). Under saline osmotic pressure, microbes inherently need to expend vast amounts of energy just to maintain cellular balance, leading to extremely poor apparent carbon use efficiency [3,11,21]. Consequently, the labile carbon mobilized by hydrolytic enzymes—likely combining rapid straw decomposition with positive priming of native SOM—was rapidly consumed via heterotrophic respiration [48]. Conversely, biochar resided largely within the recalcitrant POC and operationally defined MAOC, avoiding intensive exploitation by hydrolytic enzymes and coinciding with putative oligotrophic fungi [17,42]. Because PPO catalysis of condensed aromatic structures requires high activation energy, overall system carbon mineralization remained strongly constrained [42].

Previous studies in non-saline agricultural soils frequently report that applying exogenous organic materials, including biochar, induces an early respiratory pulse driven by minor volatile fractions or positive priming of native SOC [18,20]. In our coastal saline soil, although cumulative CO_2_ emissions under biochar exceeded CK by 58.0–102.2%, the emission curves remained flat and subdued compared with straw. Without isotopic tracing, we cannot partition this modest increase between the mineralization of minor labile biochar components and enhanced native SOM turnover following salinity alleviation; nevertheless, the absence of a respiratory surge indicates that neither extensive biochar degradation nor strong positive priming of native SOM occurred (Figure 4). This stability suggests that under saline constraints, biochar functions essentially as a “carbon desert” for energy-demanding potential copiotrophic bacteria [49,50]. Although there was a massive accumulation of total carbon in the surrounding matrix, bioavailable active sugars were virtually absent. This physical and chemical isolation of substrate availability ultimately decouples rapid soil carbon accrual from the costly heterotrophic respiratory penalty [14,17].

### 4.4. Management Implications for Soil Amelioration and Long-Term C Sequestration in Coastal Saline Soils

Restoring degraded saline agroecosystems requires reconciling the dual goals of rapidly restoring biological fertility and maximizing long-term carbon sequestration while mitigating greenhouse gas effluxes [3,5,51]. By comparing strictly homologous organic materials, this study provides clear mechanistic insights for optimizing residue management in salt-affected environments.

Direct incorporation of reed straw acts as a potent biological stimulator, rapidly elevating bioavailable nitrogen, phosphorus, potassium, and SMBC (Table 2). This priming effect is vital for breaking soil physical compaction and activating dormant biochemical turnover during early-stage reclamation [4,15]. However, the high respiratory carbon penalty and heavy allocation into unprotected POC indicate that straw-induced carbon gains are transient and vulnerable to rapid remobilization upon environmental shifts [8,14]. Conversely, while homologous biochar is conservative in providing immediate mineral nutrients, it excels at mitigating salinity, improving bulk density, buffering alkalinity, and promoting MAOC retention with minimal respiratory expenditure (Table 2, Figure 3 and Figure 4). Consequently, coastal land management should avoid monolithic residue returning and instead adopt a coordinated “straw and biochar co-application” regime. In this framework, moderate straw inputs can be applied to provide rapid biochemical priming and nutrient supply during crop establishment, while homologous biochar forms the recalcitrant MAOC backbone for salinity buffering, soil structure rehabilitation, and enhanced soil C retention.

A few limitations should be acknowledged. This study utilized controlled indoor microcosms, which successfully isolated specific mechanisms from field climatic stochasticity but omitted field-scale complexities such as drying–rewetting cycles and crop root interactions [10]. These natural disturbances continuously modulate biochar surface weathering and the priming of native organic matter [10,17]. Moreover, because the incubation was conducted on a single coastal saline soil without a non-saline soil variant or an experimental salinity gradient, the specific influence of salinity as a factor modifying C turnover mechanisms could not be experimentally isolated and remains to be investigated in future studies. Future research should therefore integrate long-term field trials with ^13^C isotopic tracing and metagenomic tools to resolve the molecular persistence and in situ organo-mineral binding kinetics of homologous amendments across coastal delta landscapes, while incorporating paired saline–non-saline soils or experimental salinity gradients to explicitly disentangle salinity effects on C turnover.

## 5. Conclusions

Using reed residues, we show that straw and biochar drive divergent carbon fates in coastal saline soil despite both alleviating salinity and increasing SOC. Straw diverted newly added C into POC (POC/SOC up to 78.2%), promoted bacterial copiotrophs (oligotroph/copiotroph ratio declined from 1.82 to 0.86), stimulated hydrolytic enzymes by factors of 4.3 to 9.1, and increased cumulative CO_2_ emissions by 375.0% to 1645.9%. Biochar maintained MAOC dominance (MAOC/SOC 62.1% to 68.0%), enriched fungal oligotrophs, suppressed hydrolytic enzymes, and caused only a modest respiratory increase (58.0% to 102.2%). Notably, because total CO_2_ efflux was measured without isotopic tracing, the low respiratory response under biochar reflects both its intrinsic aromatic recalcitrance and limited positive priming of native SOM. Statistical models indicated that these contrasting system CO_2_ responses were associated with a putative copiotrophic bacteria–hydrolytic enzyme–high respiration pathway under straw, versus an oligotrophic fungi–polyphenol oxidase–low respiration pathway under biochar. For the studied coastal saline soil, while straw alone rapidly boosts nutrient availability, it incurs substantial system respiratory C loss; a coordinated straw and biochar co-application strategy may better balance short-term nutrient supply with MAOC retention and reduced total CO_2_ emissions.

## Figures and Tables

**Table 1:** Basic physicochemical properties of reed (*Phragmites australis*) straw and its homologous biochar (pyrolyzed at 500 °C for 2 h).

Property	Unit	Reed Straw (JF)	Reed Biochar (TF)
Total C	g kg^−1^	426.5 ± 8.4	612.4 ± 11.2
Total N	g kg^−1^	8.24 ± 0.21	8.85 ± 0.24
C/N mass ratio	—	51.8 ± 1.5	69.2 ± 1.9
Ash content	%	8.24 ± 0.31	21.46 ± 0.58
pH	—	6.82 ± 0.08	7.56 ± 0.09
EC	μS cm^−1^	342.5 ± 11.4	388.6 ± 13.8

Values are means ± standard deviations (*n* = 5). Total C, total N, and ash content are expressed on a dry mass basis. C/N denotes the mass ratio of total C to total N. EC, electrical conductivity; JF, reed straw; TF, biochar derived from the same reed feedstock.

**Table 2:** Effects of straw and biochar additions on the soil physicochemical properties.

Soil Properties	CK	JF1	JF5	JF9	TF1	TF5	TF9
Temperature (°C)	26.6 ± 0.38 ^a^	25.7 ± 0.25 ^b^	25.3 ± 0.36 ^b^	25.2 ± 0.43 ^b^	25.0 ± 0.15 ^b^	25.1 ± 0.4 ^b^	25.1 ± 0.35 ^b^
Moisture (%)	21.6 ± 0.47 ^d^	24.4 ± 0.44 ^c^	28.1 ± 0.17 ^b^	32.0 ± 0.29 ^a^	27.7 ± 0.18 ^b^	27.8 ± 0.49 ^b^	25.1 ± 0.22 ^c^
Bulk density (g cm^−3^)	1.30 ± 0.03 ^a^	1.18 ± 0.02 ^b^	1.06 ± 0.02 ^c^	0.87 ± 0.01 ^d^	1.20 ± 0.02 ^b^	1.05 ± 0.02 ^c^	0.91 ± 0.02 ^d^
pH	8.5 ± 0.17 ^a^	8.2 ± 0.12 ^a^	8.3 ± 0.21 ^a^	8.2 ± 0.16 ^a^	7.8 ± 0.10 ^b^	7.6 ± 0.06 ^b^	7.3 ± 0.15 ^c^
EC (μS cm^−1^)	698.1 ± 15.9 ^a^	644.8 ± 13.6 ^b^	574.5 ± 11.9 ^c^	415.3 ± 10.5 ^e^	661.7 ± 19.7 ^b^	545.3 ± 12.8 ^d^	409.5 ± 9.2 ^e^
SOM (g kg^−1^)	11.20 ± 0.50 ^d^	14.89 ± 0.88 ^c^	39.54 ± 1.22 ^b^	59.08 ± 1.46 ^a^	17.41 ± 0.13 ^c^	37.46 ± 2.81 ^b^	51.67 ± 7.20 ^a^
SOC (g kg^−1^)	6.49 ± 0.29 ^e^	8.64 ± 0.51 ^d^	22.93 ± 1.29 ^c^	34.27 ± 0.07 ^a^	10.10 ± 0.07 ^d^	21.73 ± 1.63 ^c^	29.97 ± 4.18 ^b^
TN (g kg^−1^)	0.44 ± 0.01 ^g^	0.63 ± 0.00 ^c^	0.87 ± 0.00 ^b^	1.03 ± 0.03 ^a^	0.53 ± 0.00 ^f^	0.57 ± 0.00 ^d^	0.55 ± 0.00 ^e^
Olsen-P (mg kg^−1^)	30.72 ± 1.50 ^g^	50.44 ± 0.79 ^c^	60.76 ± 0.97 ^b^	70.62 ± 3.41 ^a^	42.17 ± 0.27 ^e^	48.36 ± 1.35 ^d^	37.34 ± 2.47 ^f^
Available K (mg kg^−1^)	107.22 ± 3.30 ^g^	251.71 ± 2.43 ^f^	343.50 ± 6.15 ^d^	425.68 ± 7.74 ^c^	322.73 ± 1.36 ^e^	621.83 ± 8.06 ^a^	574.76 ± 1.86 ^b^
NH_4_^+^-N (mg kg^−1^)	4.92 ± 0.06 ^b^	6.18 ± 0.08 ^a^	6.53 ± 0.05 ^a^	6.38 ± 0.08 ^a^	1.49 ± 0.01 ^c^	1.24 ± 0.02 ^d^	1.45 ± 0.05 ^c^
SMBC (g kg^−1^)	0.08 ± 0.00 ^e^	0.37 ± 0.01 ^c^	0.54 ± 0.02 ^b^	0.68 ± 0.01 ^a^	0.12 ± 0.01 ^e^	0.23 ± 0.00 ^d^	0.41 ± 0.01 ^c^
SMBN (g kg^−1^)	0.01 ± 0.00 ^c^	0.01 ± 0.00 ^c^	0.03 ± 0.00 ^a^	0.02 ± 0.00 ^b^	0.01 ± 0.00 ^c^	0.02 ± 0.00 ^b^	0.02 ± 0.00 ^b^

Values are means ± standard deviations (*n* = 5). Different lowercase letters within a row indicate significant differences among treatments (*p* < 0.05). Treatments: CK, unamended control; JF1, JF5, and JF9, reed straw applied at 1%, 5%, and 9% of dry soil mass, respectively; TF1, TF5, and TF9, homologous reed biochar applied at 1%, 5%, and 9% of dry soil mass, respectively. EC, electrical conductivity; SOM, soil organic matter; SOC, soil organic carbon; TN, total nitrogen; Olsen-P, available phosphorus; available K, available potassium; NH_4_^+^-N, ammonium nitrogen; SMBC and SMBN, soil microbial biomass carbon and nitrogen.

**Table 3:** Effects of straw and biochar additions on soil carbon-cycle enzyme activities.

Enzyme Activity	CK	JF1	JF5	JF9	TF1	TF5	TF9
PPO (mg d^−1^ g^−1^)	11.89 ± 0.19 ^c^	12.16 ± 0.31 ^c^	12.90 ± 0.67 ^bc^	14.34 ± 0.06 ^a^	13.03 ± 0.60 ^b^	13.18 ± 0.10 ^b^	12.33 ± 0.34 ^c^
CBH (nmol h^−1^ g^−1^)	18.43 ± 0.60 ^d^	44.69 ± 2.21 ^c^	131.16 ± 1.33 ^b^	144.18 ± 7.51 ^a^	12.83 ± 0.31 ^e^	10.95 ± 0.64 ^ef^	8.42 ± 0.45 ^f^
XYL (μmol h^−1^ g^−1^)	38.51 ± 1.85 ^d^	121.96 ± 2.80 ^c^	277.08 ± 3.33 ^b^	350.04 ± 14.87 ^a^	39.73 ± 0.21 ^d^	33.65 ± 1.37 ^e^	30.89 ± 0.44 ^e^
BG (μmol d^−1^ g^−1^)	2.74 ± 0.04 ^d^	5.19 ± 0.23 ^c^	10.51 ± 0.24 ^b^	13.04 ± 0.48 ^a^	2.71 ± 0.05 ^d^	2.96 ± 0.05 ^d^	2.60 ± 0.08 ^d^
NAG (μmol d^−1^ g^−1^)	1.16 ± 0.05 ^d^	2.34 ± 0.08 ^c^	4.25 ± 0.12 ^b^	4.96 ± 0.21 ^a^	1.00 ± 0.03 ^d^	0.97 ± 0.02 ^d^	0.65 ± 0.01 ^e^

Values are means ± standard deviations (*n* = 5). Different lowercase letters within a row indicate significant differences among treatments (*p* < 0.05). Treatments: CK, unamended control; JF1, JF5, and JF9, reed straw applied at 1%, 5%, and 9% of dry soil mass, respectively; TF1, TF5, and TF9, homologous reed biochar applied at 1%, 5%, and 9% of dry soil mass, respectively. PPO, polyphenol oxidase; CBH, cellobiohydrolase; XYL, β-D-xylosidase; BG, β-D-glucosidase; NAG, N-acetyl-β-D-glucosaminidase.

## Data Availability

The data that support the findings of this study are available from the corresponding author upon reasonable request.
